# Ventricular unloading causes prolongation of the QT interval and induces ventricular arrhythmias in rat hearts

**DOI:** 10.3389/fphys.2024.1346093

**Published:** 2024-07-03

**Authors:** Alexander Peter Schwoerer, Daniel Biermann, Heimo Ehmke

**Affiliations:** ^1^ Department of Cellular and Integrative Physiology, University Medical Center Hamburg-Eppendorf, Hamburg, Germany; ^2^ DZHK (German Center for Cardiovascular Research), Partner Site Hamburg/Kiel/Lübeck, Hamburg, Germany; ^3^ Department of Congenital and Pediatric Heart Surgery, Children’s Heart Clinic, University Heart and Vascular Center, University Medical Center Hamburg-Eppendorf, Hamburg, Germany

**Keywords:** QT interval, ventricular unloading, ventricular arrhythmias, heterotopic heart transplantation, rat heart, hemodynamic unloading, repolarization

## Abstract

**Introduction:**

Ventricular unloading during prolonged bed rest, mechanical circulatory support or microgravity has repeatedly been linked to potentially life-threatening arrhythmias. It is unresolved, whether this arrhythmic phenotype is caused by the reduction in cardiac workload or rather by underlying diseases or external stimuli. We hypothesized that the reduction in cardiac workload alone is sufficient to impair ventricular repolarization and to induce arrhythmias in hearts.

**Methods:**

Rat hearts were unloaded using the heterotopic heart transplantation. The ECG of unloaded and of control hearts were telemetrically recorded over 56 days resulting in >5 × 10^6^ cardiac cycles in each heart. Long-term electrical remodeling was analyzed using a novel semi-automatic arrhythmia detection algorithm.

**Results:**

56 days of unloading reduced left ventricular weight by approximately 50%. While unloading did not affect average HRs, it markedly prolonged the QT interval by approximately 66% and induced a median tenfold increase in the incidence of ventricular arrhythmias in comparison to control hearts.

**Conclusion:**

The current study provides direct evidence that the previously reported hypertrophic phenotype of repolarization during cardiac unloading translates into an impaired ventricular repolarization and ventricular arrhythmias *in vivo*. This supports the concept that the reduction in cardiac workload is a causal driver of the development of arrhythmias during ventricular unloading.

## Introduction

Ventricular unloading, e.g., during prolonged bed rest and ventricular assist device therapy, induces atrophic cardiac remodeling (Hill and Olson, 2008). Although it may have been described as early as in the 17th century by Riolan, cardiac atrophy remains a relatively obscure pathologic entity (Riolan, 1649; Gibson, 1898; Hellerstein and Santiago-Stevenson, 1950). In 1950, Hellerstein et al. reported several distinctive electrocardiographic features in patients with cardiac atrophy, including changes in the repolarization phase, such as a substantial prolongation of the QT interval (Hellerstein and Santiago-Stevenson, 1950). This report gave the first indication that ventricular repolarization may be affected during cardiac atrophic remodeling. Substantiating this concept, prolongation of the QT interval and an increased occurrence of ventricular arrhythmias has later been reported in other situations that are associated with cardiac atrophy like left ventricular (LV) unloading by ventricular assist device therapy, prolonged bed rest or exposure to microgravity during space flight (Douglas, 1978; Fritsch-Yelle et al., 1998; White and Averner, 2001; D'Aunno et al., 2003; Mitchell and Meck, 2004; Anzai et al., 2014; Patel et al., 2020).

Until today, however, it has never been directly addressed whether the impairment of ventricular electrophysiology is caused by ventricular unloading itself or is secondary to the underlying disease (e.g., heart failure) or its complications (e.g., electrolyte disorders, radiation). Due to inherent confounders and ethical considerations, this fundamental question cannot be resolved in patients or healthy volunteers but needs to be addressed using an experimental approach. In small animals, cardiac atrophy by ventricular unloading can be realized using the heterotopic heart transplantation (hHTx) (Ono and Lindsey, 1969; Depre et al., 1998; Schwoerer et al., 2008a; Schwoerer et al., 2008b; Didie et al., 2013; Schwoerer et al., 2013; Westhofen et al., 2019). In this model, hearts are transplanted into the abdomen of syngeneic recipients. Due to the vascular configuration, the blood flow to the graft bypasses the ventricles which results in a >50% reduction of ventricular preload and in a >80% reduction of stroke volume (Didie et al., 2013). Using this model, we have previously reported a complex remodeling of cellular electrophysiology and Ca^2+^ handling in cardiomyocytes isolated from unloaded hearts (Schwoerer et al., 2008a; Schwoerer et al., 2013). In particular, unloading was associated with a substantial downregulation of cardiac K^+^ channels, an upregulation of L-type Ca^2+^ channels and a corresponding prolongation of action potentials–all of which should increase the risk of ventricular arrythmias (Schwoerer et al., 2008a; Schwoerer et al., 2013). On the other hand, the Ca^2+^ load of the sarcoplasmic reticulum and the frequency of spontaneous Ca^2+^ releases from the sarcoplasmic reticulum were found to be substantially reduced which would predict a reduced risk of ventricular arrhythmias (Schwoerer et al., 2008a; Schwoerer et al., 2013). Overall, these studies provided a mechanistic link between ventricular unloading and ventricular repolarization on the cellular level. However, with potentially opposing effects on ventricular electrophysiology, the functional consequences on the QT interval and on the incidence of ventricular arrhythmias, however, remained unclear.

To clarify this, we now tested the hypothesis that a reduction in cardiac workload alone is sufficient to impair repolarization and to induce ventricular arrhythmias in hearts. For this, rat hearts were unloaded using the hHTX in syngeneic Lewis rats. The electrical phenotype of unloaded and control (orthotopic) hearts was monitored with telemetric ECG transponders for an unloading period of 56 days (Zimmermann et al., 2006; Nebel et al., 2013; Jelinek et al., 2018; Pecha et al., 2019). The QT interval was determined using a semi-automatic algorithm. As automatic algorithms for the detection of ventricular arrhythmias are not validated in small rodents, we developed a new semi-automatic approach. Based on >5 × 10^6^ analyzed cardiac cycles in each heart, we demonstrate that ventricular unloading alone is sufficient to prolong the QT interval and to increase the arrhythmic burden.

## Materials and methods

### Ethic statement

Experiments were carried out in accordance with institutional guidelines and were approved by local authorities (Behörde für Gesundheit und Verbraucherschutz, Freie und Hansestadt Hamburg; Germany; permit number 13/098). Furthermore, they complied with the European Convention for the Protection of Vertebrate Animals Used for Experimental and Other Scientific Purposes (Council of Europe No. 123). The authors confirm that they have taken all steps to minimize the animals’ pain and suffering. This work complies with the Journal’s policies regarding animal experiments, and the ARRIVE guidelines.

### Animal model of ventricular unloading

LV unloading was induced by hHTx in syngeneic male Lewis rats (269 ± 16 g, *n* = 16, Charles River, Sulzfeld, Germany) as previously described (Ono and Lindsey, 1969; Schwoerer et al., 2008a; El-Armouche et al., 2010; Schwoerer et al., 2013; Schaefer et al., 2016). Briefly, donor animals were anaesthetized using cardioprotective sevoflurane (4%–6%, SEVOrane^®^, Abbott Laboratories Inc., Chicago, IL, United States). 500 I.E., Heparin were injected into the vena cava. Following sternotomy, the donor’s heart was harvested, flushed with ice-cold cardioplegia solution (St. Thomas Solution I, Dr. Franz Köhler Chemie, Germany) and stored in ice-cold NaCl. The heart was then transplanted into the abdominal cavity of the recipient rat under sevoflurane anaesthesia (2.5%–3.5%). The aortic and pulmonary vessels of the donor heart were anastomosed infrarenally to the abdominal aorta and the inferior vena cava of the recipient animal, respectively, using running Prolene 8-0 sutures (Ethicon Inc., Somerville, NJ, United States). In this configuration, coronary perfusion is conserved, LV filling is substantially reduced, and LV innervation is absent, resulting in a substantial LV unloading. Spontaneous contraction returned within minutes after reperfusion and was checked regularly by palpation.

Following the transplantation procedure, telemetric ECG devices (PhysioTel^®^ TA11ETA-F10, Data Sciences International, D.S.I., St. Paul, MN United States) were implanted to record the ECG either of the orthotopic (*n* = 8) or of the transplanted heart (*n* = 8) (Zimmermann et al., 2006; Nebel et al., 2013; Jelinek et al., 2018; Pecha et al., 2019). To record the ECG of the orthotopic heart, the negative lead was placed in the area of the right shoulder and the positive lead left of the xiphoid space and caudal the rib cage. The ECG of the transplanted heart was derived with the electrodes sutured directly on the epicardium. In both configurations, the ECG approximated an Einthoven Lead II configuration.

All surgical procedures were performed under sevoflurane anesthesia, and all efforts were made to minimize suffering. Perioperative pain management of the recipient animal was performed using buprenorphine (0.04 mg/kg body weight s. c., Temgesic^®^, 30 min before the procedure, Reckitt Benckiser, Slough, Berkshire, United Kingdom) and carprofen (5 mg/kg body weight s. c., intraoperatively, Rimadyl^®^). Postoperatively, the animals were treated with buprenorphine (0.04 mg/kg body weight s. c, three days every 8 h) and metamizole (100 mg/kg bodyweight p. o., seven days postoperatively, Novalgin^®^).

Due to the syngeneic nature of the Lewis rats, no immunosuppression had to be performed. For all experiments, orthotopic hearts served as corresponding controls. Following the observation period of 56 days, the transplanted (unloaded) and the orthotopic (control) hearts of the recipient animal were removed in deep anaesthesia (sevoflurane 5%–7% in combination with buprenorphine treatment 30 min before intervention).

### ECG monitoring and ECG analysis

The ECG was recorded using Dataquest A.R.T. (v. 4.3, D.S.I.) or Ponemah (v. 6.0, D.S.I.) with a recording sampling rate of 1,000 Hz and no additional filters. Animals were housed in individual cages on a receiver plate and allowed free access to food and water. Day-night rhythm was established with daytime (lights on) between 07:00 a.m.–07:00 PM. Room temperature and humidity were controlled at 20°C–22°C and 40%–60%, respectively. Offline ECG analysis was performed using Dataquest A.R.T. (v. 4.3, D.S.I.), Ponemah (v. 6.0, D.S.I.) and ecgAUTO (v.2.5.1.35, emka technologies). ECG analysis was performed by an ECG expert who was not informed about the details of the study design and was not involved in practical procedures associated with the experiments and the ECG recordings, and thus was blinded to the specific experimental groups. The heart rate (HR) and QT interval were calculated on a beat-to-beat basis and were averaged over 1 minute. QT intervals were determined using manually designed libraries with heart-specific ECG waveforms in ecgAUTO. Waveform libraries included *n* = 42 ± 24 (range 10–88) representative waveforms per heart. There was no difference between the two groups in the number of templates per heart. Labelling of each ECG segment was manually performed. The use of specific waveform libraries for each heart (and not for each experimental group) was necessary to account for waveform differences in the ECGs and to facilitate tolerable computing times. For calculation of QT intervals, the ECG waveforms were matched to the templates in the library and the best fitting template was used by the software to calculate the QT interval. For validation of QT data generated by this approach, waveforms were regularly also analyzed manually using printouts and Ponemah.

Abnormal ventricular beats were identified based on irregularities in consecutive RR intervals consistent with ventricular arrhythmias (e.g., premature beats, postextrasystolic pause). To further increase the detection sensitivity, an ECG waveform analysis using heart-specific waveform libraries was performed. Arrhythmias were classified according to the guidelines of The Lambeth Conventions (Walker et al., 1988; Zimmermann et al., 2006; Nebel et al., 2013; Jelinek et al., 2018; Pecha et al., 2019). Controversial ECG segments were independently classified and consented by three ECG experts.

Premature ventricular contractions (PVC) were identified by the presence of at least two of the following three criteria.

1) atypical QRS complex or QRS vector with alteration of the T-wave
2) absence of detectable P-wave or atrioventricular dissociation
3) abbreviated RR interval before and compensatory pause following the beat

Two or three consecutive PVCs were defined as a salve, whereas a run of four or more consecutive PVCs as a ventricular tachycardia (VT). Ventricular fibrillation (VF) was identified by the absence of distinguishable individual QRS complexes and the absence of isoelectric phases.

QT variance (QTV) and HR variance (HRV) were calculated as the square of the standard deviation. The QT variability was assessed using the QT variability index (QTVI): $\text{QTVI}=\log\frac{\left(\frac{\text{QTV}}{\text{mean}\;{\text{QT}}^{2}}\right)}{\left(\frac{\text{HRV}}{\text{mean}\;{\text{HR}}^{2}}\right)}$ (Dobson et al., 2013).

### Statistical analysis

Data management was performed using Microsoft Excel (Office 365, Microsoft, Redmond, MN, United States). Statistical analysis was performed with Graph Pad Prism (v. 9.3.0, Graphpad, La Jolla, CA, United States). In the main text and figure legends, all data are given as mean ± SD. In the figures, data are given as mean ± SD, or median ± interquartile ranges, as indicated. Gaussian distribution was tested using D'Agostino and Pearson omnibus normality test. Parametric data were compared using unpaired Student’s t-test, or repeated measures ANOVA with Sidak *post hoc* tests, corrected for multiple comparisons. Nonparametric data were compared using unpaired Mann-Whitney test. Statistical significance was defined as *p* < 0.05.

## Results

**FIGURE 1:**
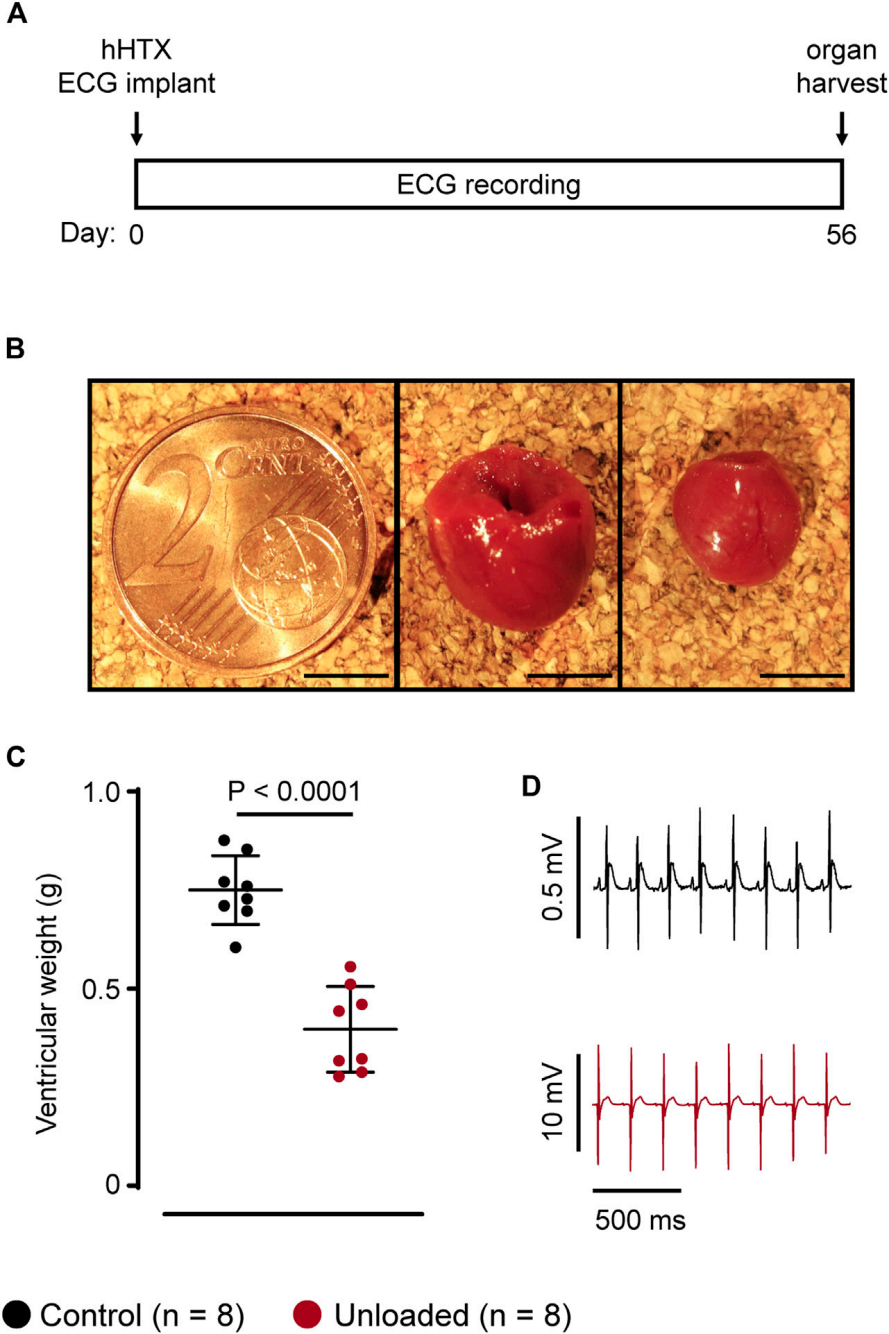
Study design and animal model. **(A)**, study design. Heterotopic heart transplantation (HTX), and implantation of telemetric ECG transponders (ECG) was performed at day 0. ECGs of either control or unloaded hearts were recorded over 56 days. Hearts were explanted at day 56. **(B)**, representative control heart (middle panel) and unloaded heart (right) harvested at day 56 with a two-cent piece (left) for size comparison. Scale bars represent 5 mm. **(C)**, average ventricular weights (mean ± SD) of control (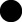, 750 ± 87 mg, *n* = 8) and unloaded hearts (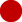, 397 ± 109 mg, *n* = 8, *p* < 0.0001) at day 56. Statistical significance was calculated using a parametric unpaired Student’s *t*-test. **(D)**, representative ECG recording of a control (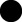) and an unloaded (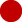) heart at comparable heart rates (approximately 320 bpm). The higher signal amplitude, e.g., of the QRS complex, and the better signal to noise ratio in unloaded hearts is due to the epicardial placement of the electrodes.

LV unloading was successfully induced by hHTx in 16 male Lewis rats. All animals survived the transplantation procedure and the observation period of 56 days (Figure 1A). Illustrating the degree of unloading, the LV weight of the unloaded heart was reduced by approximately 50% (Figures 1B, C; unloaded: 397 ± 109 mg, *n* = 8 vs. control: 750 ± 87 mg, *n* = 8, *p* < 0.0001), which is well compatible with our previous reports using an unloading period of 14 days (Schwoerer et al., 2008a; Schwoerer et al., 2008b; El-Armouche et al., 2010; Schwoerer et al., 2013). One-lead ECG recordings of either the control (*n* = 8) or of the unloaded (*n* = 8) hearts were performed over the whole period and yielded stable signals with high signal to noise ratios (Figure 1D).

**FIGURE 2:**
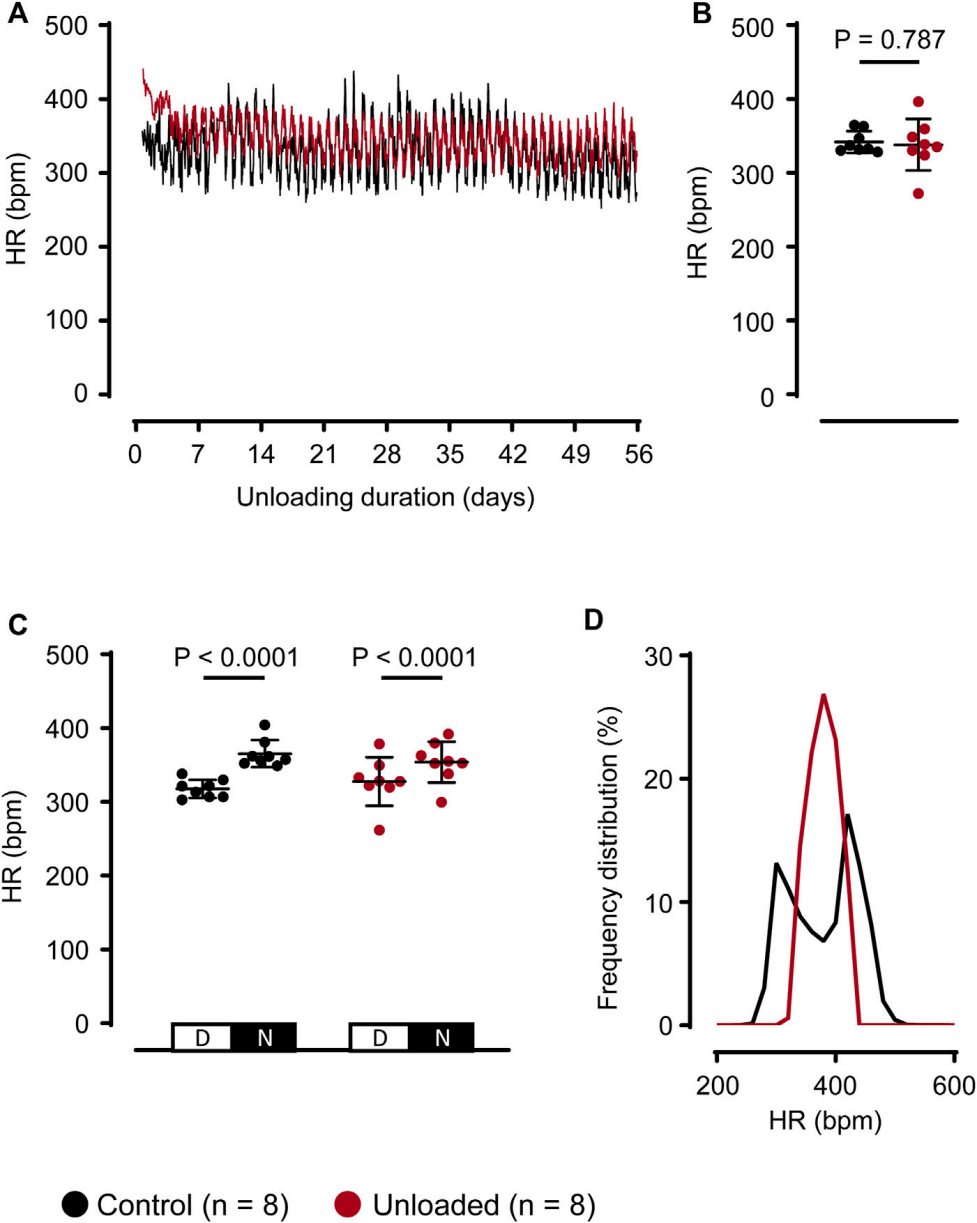
Unloading does not affect average heart rate regulation. **(A)**, representative HRs of a control (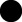) and an unloaded (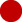) heart over 56 days. For means of clarity, HR values are averaged over 15 min **(B)**, average HR (mean ± SD) of control (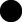, 342 ± 15 bpm, *n* = 8) and unloaded hearts (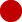, 338 ± 35 bpm, *n* = 8, *p* = 0.787) over 56 days. Statistical significance was calculated using a parametric unpaired Student’s t-test. **(C)**, average HR (mean ± SD) during daytime (D, 07:00 a.m.–07:00 p.m.) and nighttime (N, 07:00 p.m.–07:00 a.m.) of control (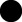, *n* = 8) and unloaded hearts (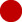, *n* = 8) over 56 days. Statistical significance was calculated using a repeated measures ANOVA with Sidak *post hoc* tests, corrected for multiple comparisons.**(D),** relative frequencies (bin width = 20) of occurring HR, calculated over 56 days for all control (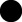, *n* = 8) and unloaded (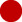, *n* = 8) hearts. Bpm, beats per minute.

Following the operational procedure, individual day-night rhythms with stable HR regulations returned within the first postoperative week in all animals. Thereafter, all control and unloaded hearts displayed visible circadian rhythms indicating intact HR regulation and recovery of the recipient animal (exemplary time course: Figure 2A). The denervation and the mechanical unloading may influence the HR of the unloaded hearts. Contrasting this expectation, the mean HR of unloaded and control hearts were similar (Figure 2B gives mean HR values over 56 days; control: 342 ± 15 bpm, *n* = 8 vs. unloaded: 338 ± 35 bpm, *n* = 8, *p* = 0.787). Compatible with a circadian HR regulation, HR was significantly higher during the nighttime than during the daytime in both, control and unloaded hearts (Figure 2C). The range of occurring HRs, however, was broader in control hearts and also exhibited a bimodal distribution (Figure 2D).

**FIGURE 3:**
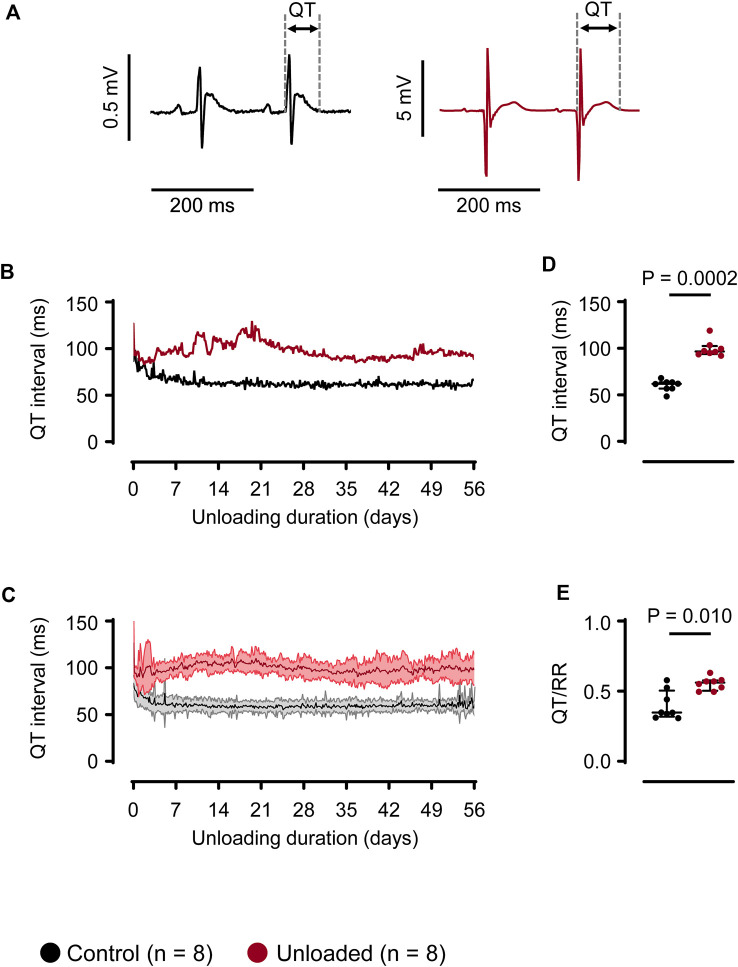
Unloading induces prolongation of the QT interval. **(A)**, representative QT intervals of a control (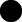) and unloaded (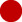) heart. **(B)**, representative time course of QT intervals of one control (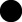) and one unloaded (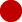) heart. For means of clarity, QT values are averaged over 15 min **(C)**, time course of QT intervals (mean ± SD) of all control (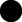, *n* = 8) and all unloaded (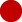, *n* = 8) hearts. For means of clarity, QT values are averaged over 15 min **(D)**, average duration of QT intervals of control (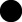, 60 ± 6 ms, *n* = 8) and unloaded hearts (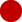, 99 ± 9 ms, n = 8, *p* = 0.0002) calculated over 56 days. **(E),** QT/RR ratio of control (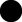, 0.41 ± 0.11 ms, *n* = 8) and unloaded hearts (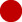, 0.55 ± 0.04 ms, *n* = 8, *p* = 0.0281), calculated using the RR intervals immediately preceding the QT intervals. **(D, E)**, lines and error bars denote median with interquartile range. Statistical significance was calculated using a nonparametric unpaired Mann-Whitney test.

The QT intervals were assessed using a waveform-based approach. For this, ECG libraries with manually annotated waveforms were specifically generated for each heart (Figure 3A) Using these libraries, the QT intervals were determined for each recorded heartbeat over 56 days Figure 3B depicts a representative time course for one control and one unloaded heart. In the immediate postoperative phase, QT intervals were prolonged in both, control and unloaded, hearts. QT intervals regularly exceeded 75 ms in control and 100 ms in unloaded hearts. This initial prolongation most likely illustrates the intra- and perioperatively applied drugs and the severe decrease of the abdominal temperature during the operation. Following the recovery of the animals, the QT interval of all control hearts stabilized at a length of approximately 60 ms (Figure 3C gives mean values for all hearts). In unloaded hearts, however, the ventricular repolarization was impaired, resulting in a mean QT interval of approximately 100 ms (Figure 3C). This prolongation of the QT interval was visible in all hearts following 4 days of unloading, without systematic further progression over the observation period. Averaged over 56 days, the mean QT interval was significantly longer in unloaded than in control hearts (Figure 3D; control: 60 ± 6 ms, *n* = 8 vs. unloaded: 99 ± 9 ms, *n* = 8, *p* = 0.0002). To exclude that this prolongation of the QT interval is caused by an adaptation to alterations in the intrinsic HR, which are not reflected in the average beating rates, the relative length of the QT interval was also determined. In the absence of QT correction formulas that are validated for the high beating rates of rat hearts, we used the QT/RR index. Consistent with the effect on the absolute length of the QT interval, the mean QT/RR index was substantially increased in the unloaded hearts (Figure 3E; control: 0.40 ± 0.11, *n* = 8 vs. unloaded: 0.55 ± 0.04, *n* = 8, *p* = 0.0104).

**FIGURE 4:**
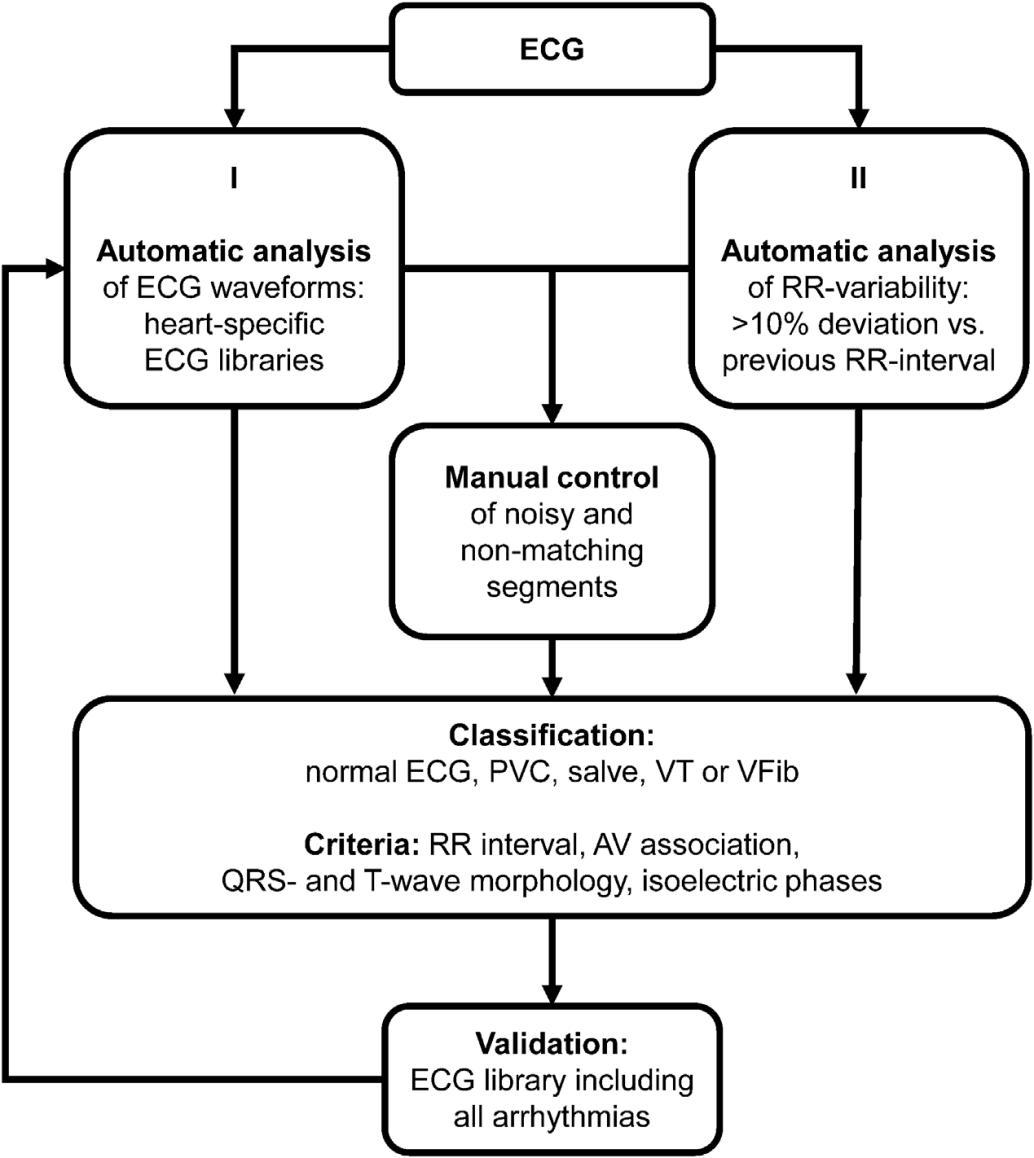
Arrhythmia analysis. Algorithm for classification of ventricular arrhythmias. PVC, premature ventricular contraction; VT, ventricular tachycardia; VFib, ventricular fibrillation; AV, atrioventricular.

To investigate whether the impairment of repolarization translates into an increased arrhythmic load, the ECGs of the complete observation period (56 days) were analyzed for ventricular arrhythmias. In rodents, fully automatic arrhythmia detection algorithms are not validated for long-term ECG analysis. The relatively high beating rates in combination with the remarkable electrical stability of the hearts challenges studies addressing arrhythmic event rates in these animals. To ensure high detection rates for ventricular arrhythmias, we used a semi-automatic approach with two independent detection strategies (Figure 4). I), a waveform library was generated with typical normal ECG waveforms of the individual heart. The recorded ECGs were annotated and classified using these libraries. Segments that could not be matched with the ECG templates, e.g., due to noise or arrhythmic events, were manually controlled by at least one ECG expert. II), based on automatic RR-detection, ECG segments with irregular RR intervals (deviation of the RR interval >10% compared to the previous RR interval) were automatically selected. All ECG segments in question were then examined and classified by at least one ECG expert. To identify potentially undetected arrhythmias and to validate the previous results, the complete ECG was scanned using an ECG library consisting of the waveforms of all normal heartbeats and of all detected arrhythmias. ECG unsuitable for analysis due to inadequate signal quality (e.g., due to loss of ECG recordings during cleaning of the cage) was <0.1% of the recorded ECGs.

**FIGURE 5:**
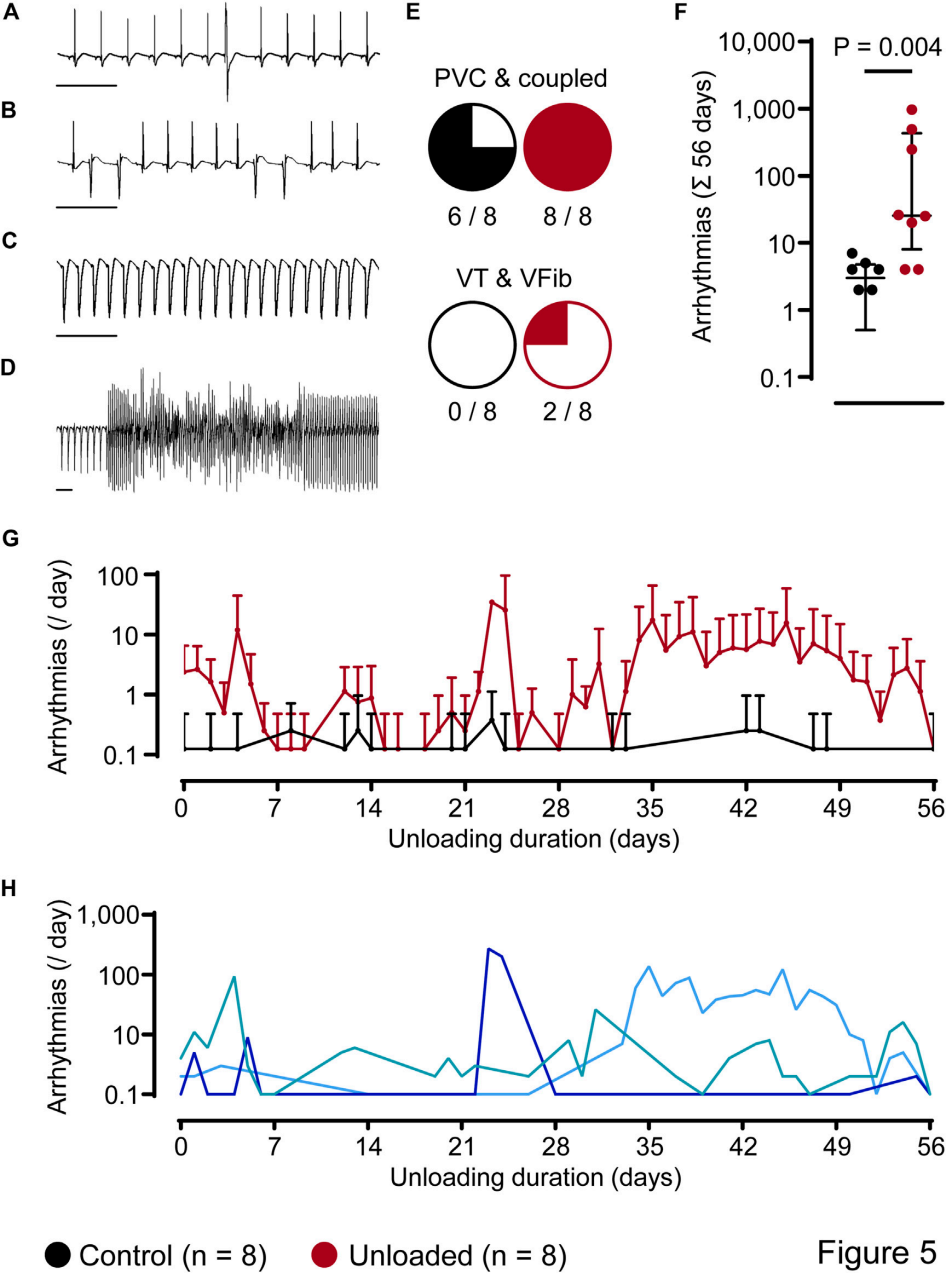
Unloading induces ventricular arrhythmias. **(A–D)**, representative ECG segments with premature ventricular contraction [PVC, **(A)]**, couplets **(B)**, ventricular tachycardia [VT, **(C)**] and ventricular fibrillation [VFib, **(D)**]. **(E)**, number of hearts with different subtypes of arrhythmias. **(F)**, total amount of ventricular arrhythmias, independent of the subtype, over 56 days, of control (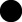, 3 ± 2 events/56 days, *n* = 8) and unloaded hearts (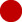, 224 ± 349 events/56 days, *n* = 8, *p* = 0.0039). Values of two control hearts that did not exhibit arrhythmias are not plotted on the logarithmic scale. Statistical significance was calculated using a nonparametric unpaired Mann-Whitney test. Lines and error bars denote median with interquartile range. **(G)**, time course of the average incidence of ventricular arrhythmias per day (mean ± SD) detected in all control (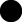, *n* = 8) and all unloaded hearts (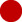, *n* = 8). On days without detected arrhythmias, mean and SD values of the control group are not plotted on the logarithmic scale. **(H)**, representative time courses of unloaded hearts (arrhythmias per day). One heart (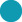) displayed an early onset and continuous arrhythmic burden. In another heart (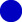), the arrhythmic burden was mainly increased in a time period of 22–28 days of unloading. In the third example (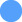), we detected a late onset of arrhythmias starting at 28 days of unloading.

ECG analysis over 56 days revealed ventricular arrhythmias that were classified as singular premature ventricular contractions (PVC, Figure 5A), coupled beats (e.g., couplets and triplets, Figure 5B), episodes of ventricular tachycardia (VT, Figure 5C) and ventricular fibrillation (VFib, Figure 5D). In general, simple ventricular arrhythmias (PVCs and coupled beats) were the most abundant arrhythmic events and could be detected in 6/8 control hearts and 8/8 unloaded hearts (Figure 5E). Furthermore, three episodes of non-sustained VT and one episode of VFib were detected in 2/8 unloaded hearts (Figure 5E). When calculated over the observation period of 56 days, the total amount of ventricular arrhythmias was significantly higher in unloaded than in control hearts (Figure 5F; control: 3 ± 2 events/56 days, *n* = 8 vs. unloaded: 224 ± 349 events/56 days, *n* = 8, *p* = 0.0039). In both groups, ∼2/3 of all arrhythmias were observed during the nighttime (07:00 p.m.–07:00 a.m.; control hearts: 66% ± 0.3%; unloaded hearts: 69% ± 0.3%).

**FIGURE 6:**
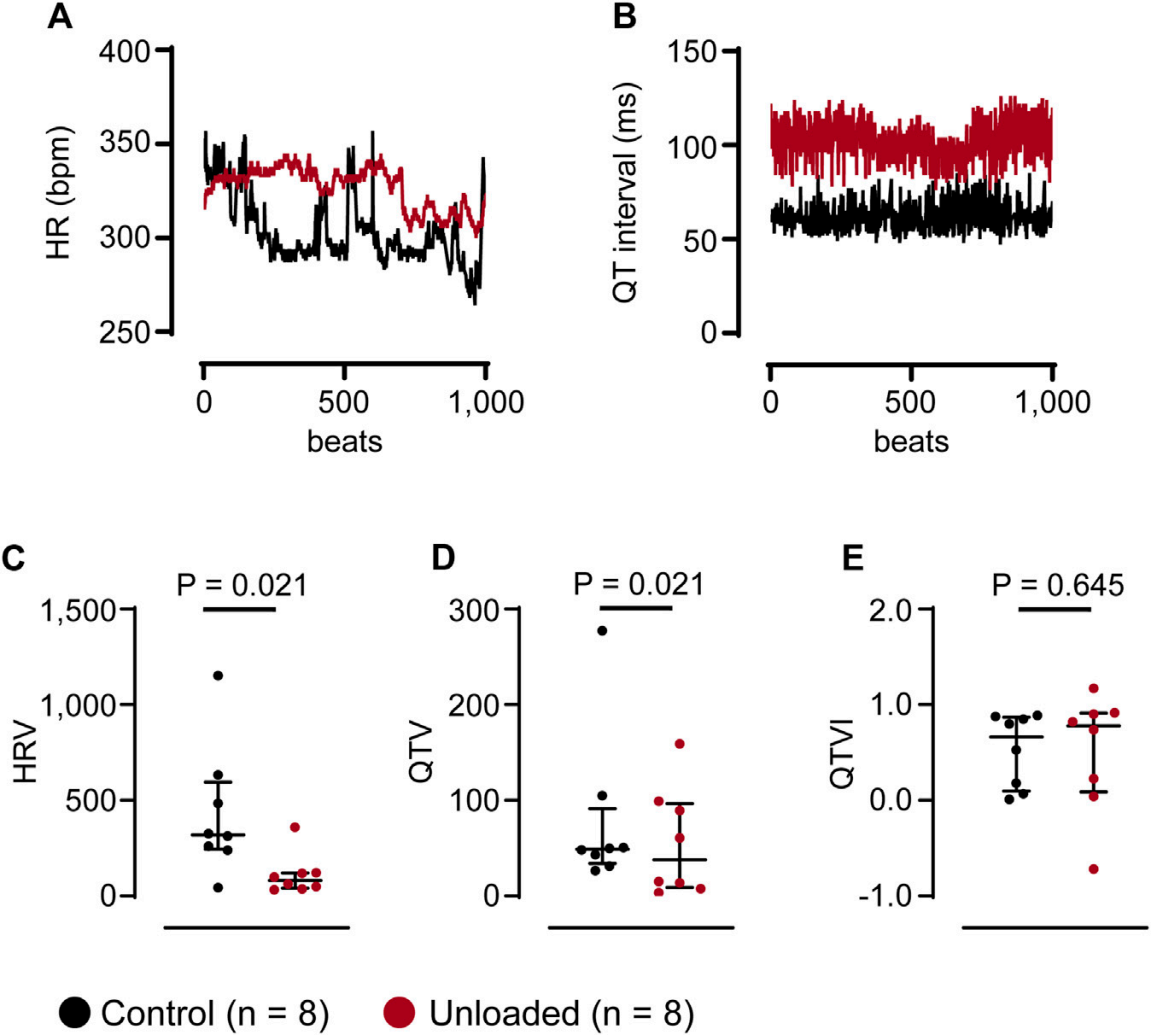
Unloading does not affect the QT variability index. **(A, B)**, representative HR and QT data of 1,000 consecutive beats, extracted at the end of the unloading period (day 56) from one control (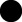) and one unloaded (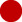) heart. The *Y*-axis of the HR was truncated to give a better impr **(C–E)**, average HRV, QTV and QTVI of control (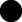, *n* = 8) and unloaded hearts (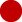, *n* = 8) calculated from time series as given in **(A, B)**. **(C–E)**, lines and error bars denote median with interquartile range. Statistical significance was calculated using a nonparametric unpaired Mann-Whitney test.

Arrhythmic events did not occur in clear temporal relation to the surgical procedure. Figure 5G depicts the time course of the mean daily arrythmic burden averaged in all hearts. Ventricular arrhythmias rather occurred with a high interindividual variance. While some unloaded hearts developed ventricular arrhythmias directly following the transplantation, others did so after three or more weeks (representative examples of three individual animals with different time courses are given in Figure 5H). Cardiac arrhythmias may be caused by an increased variability in ventricular repolarization. This can be assessed using the QT variability index (QTVI), which relates the QT variability (QTV) to the HR variability (HRV) (Dobson et al., 2013). To assess a potential role in the unloaded hearts, HRV and QTV was calculated from 1,000 consecutive beats (Figures 6A, B) at the end of the unloading period (day 56). While HRV (Figure 6C) and QTV (Figure 6D) was significantly lower in unloaded hearts, the QTVI was identical in control and unloaded hearts (Figure 6E).

## Discussion

In the current study we provide evidence for the concept that a decrease in cardiac workload is sufficient to impair repolarization and to induce ventricular arrhythmias.

### Prolongation of the QT interval

Following the transplantation and the perioperative recovery, the QT interval of unloaded hearts increased by approximately 60%. The QT interval reflects the duration of ventricular action potentials, in particular the balance between depolarizing and repolarizing ionic currents. Using the same animal model, we previously showed that cardiac K^+^ currents (I_sus_ and I_to_) are substantially downregulated and Ca^2+^ currents (I_Ca,L_) are upregulated (Schwoerer et al., 2008a; Schwoerer et al., 2013). These alterations in currents were paralleled by changes in the expression of underlying channel subunits, and on a single cell level, a pronounced prolongation of action potentials. The current study now demonstrates that the electrophysiological remodeling detected in isolated cardiac myocytes translates into a relevant prolongation of the QT interval *in vivo*. In several species, the length of the QT interval is regulated inversely to the HR (Hayes et al., 1994). In rodents, the dependence of the QT interval of HR is debatable (Mulla et al., 2022). This raises the possibility that the prolongation of the QT interval could also be a functional consequence of bradycardia in unloaded hearts. In unloaded hearts, however, the HRs were highly comparable to the HRs of control hearts, and also displayed synchronized circadian rhythms. To account for the remaining differences in HR regulation and the possibility that the HR/QT correlation is altered in the unloaded hearts, the QT intervals were corrected for the prevailing HR using the QT/RR ratio which was also significantly increased in unloaded hearts. This confirms that the prolongation of the QT interval is not secondary to a bradycardia.

### Ventricular arrhythmias

Healthy hearts of small animals are electrically highly stable and only rarely present ventricular arrhythmias, which challenges automatic arrhythmia detection algorithms of ECG software (Rossi et al., 2014; Pecha et al., 2019). Validated algorithms for long-term analysis in rodents have not been available at the time of the current study. We, therefore, employed a newly developed approach to detect ventricular arrhythmias in control and unloaded hearts. Using this approach, only very few arrhythmic events (<10 per animal), and no clinically relevant arrhythmias (e.g., ventricular tachycardia or ventricular fibrillation), were detected in control hearts during the observation period of 56 days. Unloaded hearts, on the other hand, displayed an approximately tenfold increase in the median incidence of spontaneous ventricular arrhythmias, including several episodes of potentially life-threatening ventricular tachycardia or fibrillation.

The increased arrhythmic burden of unloaded hearts seems to be primarily a consequence of the prolongation of the QT interval. On cellular level, the impairment of repolarizing K^+^ currents (Schwoerer et al., 2008a), and the increased systolic Ca^2+^ influx during the prolonged plateau phase increase the net amount of Ca^2+^ extruded *via* the Na^+^ Ca^2+^ exchanger (Schwoerer et al., 2013). This depolarizing mechanism, in turn, increases the risk for early afterdepolarizations. Increased spontaneous Ca^2+^ releases from the sarcoplasmic reticulum due to an increased Ca^2+^ load or a reduced threshold for Ca^2+^ releases (Kashimura et al., 2010; Eisner, 2014), as well as an increased automaticity are unlikely to contribute to the generation of arrhythmias. We have previously shown that the Ca^2+^ handling is shifted to an anti-arrhythmogenic phenotype (Schwoerer et al., 2008b; Schwoerer et al., 2013), and we could not detect any evidence for electrical immaturity which may cause automaticity, such as a re-expression of T-type Ca^2+^ currents or funny currents (Schwoerer et al., 2008a). Further mechanisms have previously been associated with cardiac unloading which may affect the conduction system, or cellular depolarization or repolarization. These may also contribute to the arrhythmias on the basis of the robust prolongation of the QT interval. This includes QT variability, greater dispersion of repolarization, T-wave alternans or other, more diffuse changes (Hellerstein and Santiago-Stevenson, 1950; D'Aunno et al., 2003; Sakowski et al., 2011; Popova and Rusanov, 2023).

In previous studies using the hHTx in rats as an animal model for ventricular unloading, a steady state of remodeling was often assumed to be reached after a period of two weeks of unloading (Depre et al., 1998; Schwoerer et al., 2008b; Schwoerer et al., 2013). In the current study, this assumption could be confirmed for the prolongation of the QT interval which represents the molecular remodeling. In contrast, major inter-individual differences were observed in the spatial distribution of ventricular arrhythmias over the period of 56 days, without any evidence for a systematic time dependency. Thus, a study focussing on the first two weeks of unloading would potentially miss a statistically significant difference in the occurrence of functional, rare events such as ventricular arrhythmias.

### Translational implications

The main result of the current study may translate into cardiovascular healthy patients experiencing reductions of cardiac workload during exposure to microgravity (Fritsch-Yelle et al., 1998; White and Averner, 2001; D'Aunno et al., 2003; Anzai et al., 2014). Astronauts, especially during long term flights, face an increased risk for cardiac arrhythmias (Fritsch-Yelle et al., 1998; Anzai et al., 2014; Zhu et al., 2015; Popova and Rusanov, 2023). This is currently regarded by the NASA as one of the leading cardiovascular risks for long term space flight, e.g., to Mars (Patel et al., 2020; Siddiqui et al., 2024). The mechanisms inducing cardiac arrhythmias in astronauts, i.e., the acute triggers, have not been clearly identified yet (Popova and Rusanov, 2023; Siddiqui et al., 2024). Amongst others, cardiac deconditioning, electrolyte disorders, fluid shifts, psychological stress, altered levels of catecholamines, cosmic radiation, bradycardia, T-wave alternans, the use of QT prolonging drugs or heavy work are currently discussed to contribute (Anzai et al., 2014; Zhu et al., 2015; Popova and Rusanov, 2023; Siddiqui et al., 2024). Our current study suggests that the mechanical unloading due to microgravity during space travel may also induce a cardiac remodeling that could causally contribute to the increased arrhythmic load, implying potential new targets for the prevention.

The current results may also be relevant in other conditions with reduced cardiac pre- and/or afterload, e.g., prolonged bed rest (Perhonen et al., 2001a; Perhonen et al., 2001b; Dorfman et al., 2007; Kozakova et al., 2011). Patients undergoing prolonged bed rest also experience LV unloading leading to cardiac atrophy which can be reversed by exercise training (Perhonen et al., 2001b). Whether this is also associated with prolongation of the QT interval and increased risk for arrhythmias is currently unclear. Arguably, however, the mechanisms leading to cardiac atrophy may be similar to those during microgravity. In heart failure patients undergoing left ventricular mechanical support, newly occurring ventricular arrhythmias are a matter of concern (Harding et al., 2005; Bedi et al., 2007; Andersen et al., 2009; Pedrotty et al., 2013; Shirazi et al., 2013; Galand et al., 2018; Martins et al., 2019). In addition to the reduced cardiac workload during assist device therapy, however, other mechanisms may be more relevant to the induction of arrhythmias in this setting. Most importantly, cardiac electrophysiology is affected by the underlying heart disease, direct effects of the pump, scar tissue and suction events (Pedrotty et al., 2013). To allow translation of the current results into these patients, unloading of failing hearts is, therefore, necessary.

In experimental approaches, an acute increase in stretch has been shown to induce cardiac arrhythmias by providing substrates and/or triggers (e.g., (Levine et al., 1988; Franz et al., 1989; Taggart et al., 1992b)). This may be mediated by stretch activated channels (Ott and Reiter, 1997). In line with the concept of mechanoelectric feedback, this has led to the assumption that acute unloading may have antiarrhythmic effects. While there is scarce evidence for this hypothesis, it is supported by clinical observations that acute unloading, e.g., during a cardiopulmonary bypass, during intraaortic balloon counterpulsation or during a Valsalva manoeuvre, may terminate arrhythmias (Taggart et al., 1988; Taggart et al., 1992a; Fotopoulos et al., 1999). This seems to be in contrast to the results of the current study. However, unloading using the hHTX induces a cellular remodeling, i.e., of cardiac repolarization. Thus, while positive effects on cardiac arrhythmias, potentially with the involvement of stretch activated channels, may be induced by acute unloading, the remodeling during chronic unloading rather seems to be arrhythmogenic.

### Degree of unloading

In the present study, we performed hHTX, a well characterized experimental model (Fu et al., 2016; Benke et al., 2017), to induce complete ventricular unloading. Partial unloading may have smaller effects, e.g., on the level of atrophy, fibrosis, Ca^2+^ handling, contractility, and beta-receptor density. (Didie et al., 2013; Fu et al., 2016; Benke et al., 2017). Other effects may be provoked to a similar degree, as they appear to be independent of the degree of unloading, e.g., the activation of cytokines and metalloproteinases (Didie et al., 2013; Fu et al., 2016; Benke et al., 2017). We induced complete unloading to allow for a strong stimulus for cellular remodeling to clarify whether unloading impairs repolarization and induces arrhythmias at all. The degree of unloading in the present study is comparable to that found in patients receiving a HeartMate system (Radovancevic et al., 1992). However, the degree of unloading observed in other clinical settings, e.g., bedrest, and during long term space travel should be lower in most cases. Further studies are therefore needed to define the dose-dependency of electrophysiological remodeling and to determine potentially relevant thresholds for the induction of arrhythmias.

### Unloading and heart failure

It is surprising that cardiac remodeling during unloading shares properties with remodeling during heart failure, although the overall consequences are different. This includes similar effects on cellular electrophysiology, on autophagic and apoptotic pathways, on extracellular matrizes and on fetal like gene expression profiles. On the other hand, heart failure and unloading also have substantial opposite effects on cellular (e.g., EC coupling, cell size) and on ventricular properties (e.g., wall stress, left ventricular geometry, cardiac function), and also on systemic homeostasis (e.g., oxygen supply in the body, activation of sympathetic nerve activity and humoral systems) (Fu et al., 2016; Billman, 2020). While the biological relevance of the specific similarities and differences between heart failure and unloading remain to be clarified, the present study further supports the concept that both conditions have similar overall effects on the cardiac electrophysiology.

## Conclusion

In conclusion, our current study shows that the cellular remodeling of cardiac ion channels and of the sarcoplasmic reticulum in the animal model of hHTX translates into a prolongation of the QT interval and ventricular arrhythmias. This provides evidence that a reduced ventricular workload is sufficient to impair ventricular electrophysiology. In clinical conditions which are associated with ventricular unloading, this finding may be relevant as a therapeutic target.

## Data Availability

The raw data supporting the conclusion of this article will be made available by the authors, without undue reservation.
